# Single coronary artery complicated with type A aortic dissection: a case report

**DOI:** 10.1186/s44215-025-00204-7

**Published:** 2025-03-31

**Authors:** Yuji Naito, Fumitaka Suzuki, Tatsuya Murakami

**Affiliations:** Department of Thoracic Surgery, Asahikawa City Hospital, 1-65, Kinsei-cho, Asahikawa, Hokkaido 070-8610 Japan

**Keywords:** Coronary artery anomaly, Single coronary artery, Dissecting aortic aneurysm

## Abstract

Congenital coronary artery anomalies complicated with aortic dissection are rare. We experienced a patient with a single coronary artery presenting with a type A dissecting aortic aneurysm. A 77-year-old woman who experienced sudden back pain and was diagnosed with type A acute aortic dissection was initially treated conservatively because the false lumen thrombosed entirely. Computed tomography taken during 5 weeks of hospitalization incidentally revealed an anomalous single coronary artery arising from the left sinus of Valsalva, and the right coronary artery orifice was absent. One month after being transferred to another institution for rehabilitation, she was reintroduced to us for re-dissection of the aorta. An urgent operation involving aortic arch replacement was performed. There was a solitary coronary artery orifice at the left sinus of Valsalva and no ostium at the right sinus. The postoperative course was uneventful, and the patient was discharged 14 days after the surgery. Only 3 cases involving a single coronary artery complicated with dissecting aortic aneurysm have been reported previously.

## Background

Single coronary artery is a rare congenital coronary artery anomaly with a reported prevalence of 0.024% to 0.066% [1–4]. The anomaly is usually discovered incidentally and does not necessarily indicate treatment, but intervention is taken into consideration when coronary ischemia is suspected due to atherosclerotic changes or anatomical reasons. We experienced a rare case in which computed tomography during conservative therapy for acute type A aortic dissection with a thrombosed false lumen incidentally revealed the anomaly, and aortic surgery was performed in the chronic phase.

## Case presentation

**Fig. 1:**
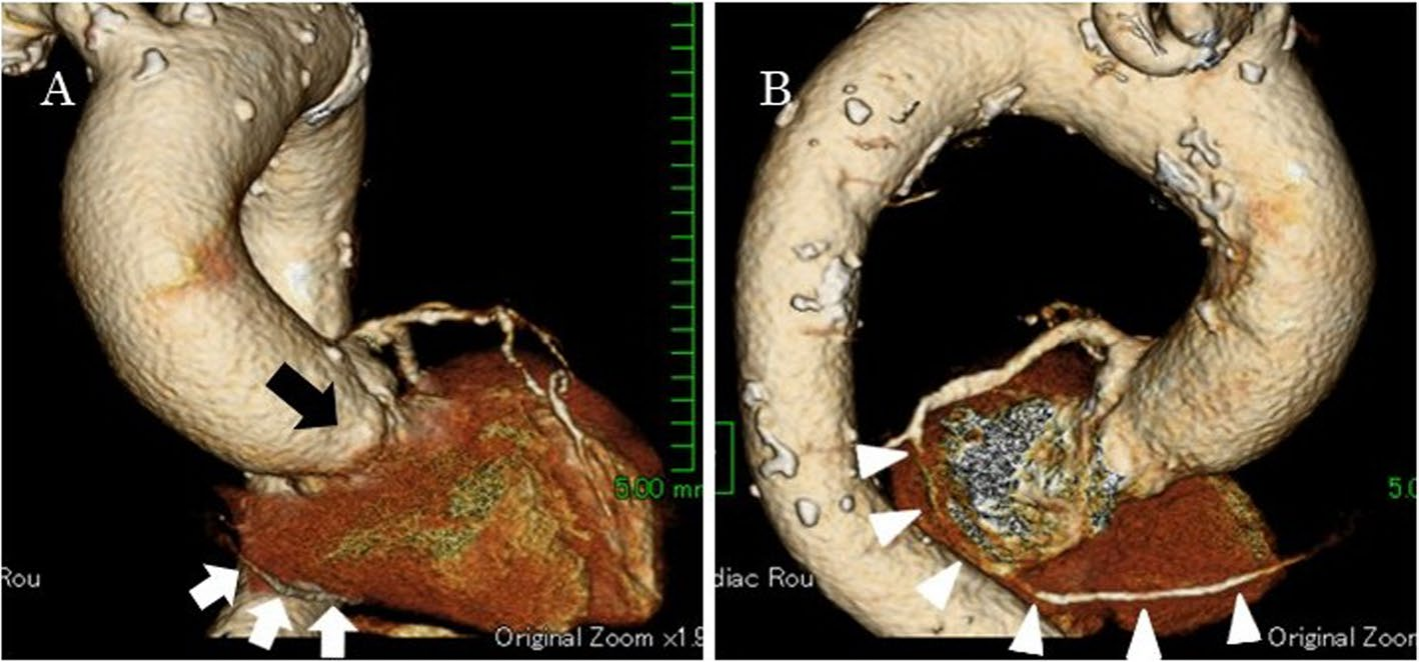
3D computed tomography image taken during conservative therapy. **A** Front view. The white arrows indicate the coronary artery continuing from distal segment of the left circumflex artery. The right coronary artery is absent at the Valsalva sinus, as indicated by the black arrow. **B** The arrowheads show the distal segment of the left circumflex artery continuing to the right coronary artery at the atrioventricular groove

**Fig. 2:**
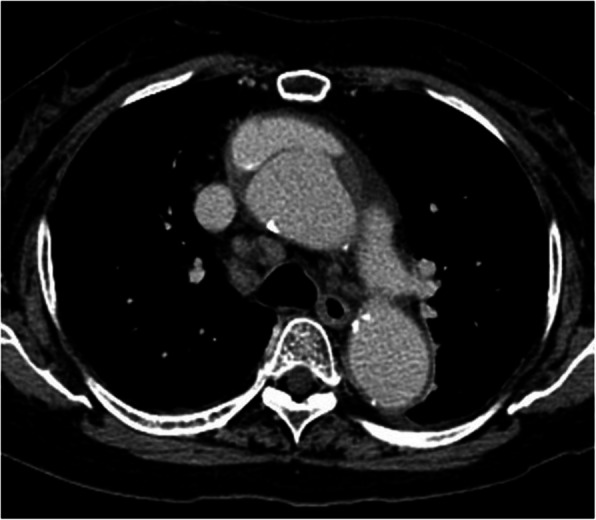
A computed tomography image before an urgent operation showing a patent false lumen at the ascending aorta

**Fig. 3:**
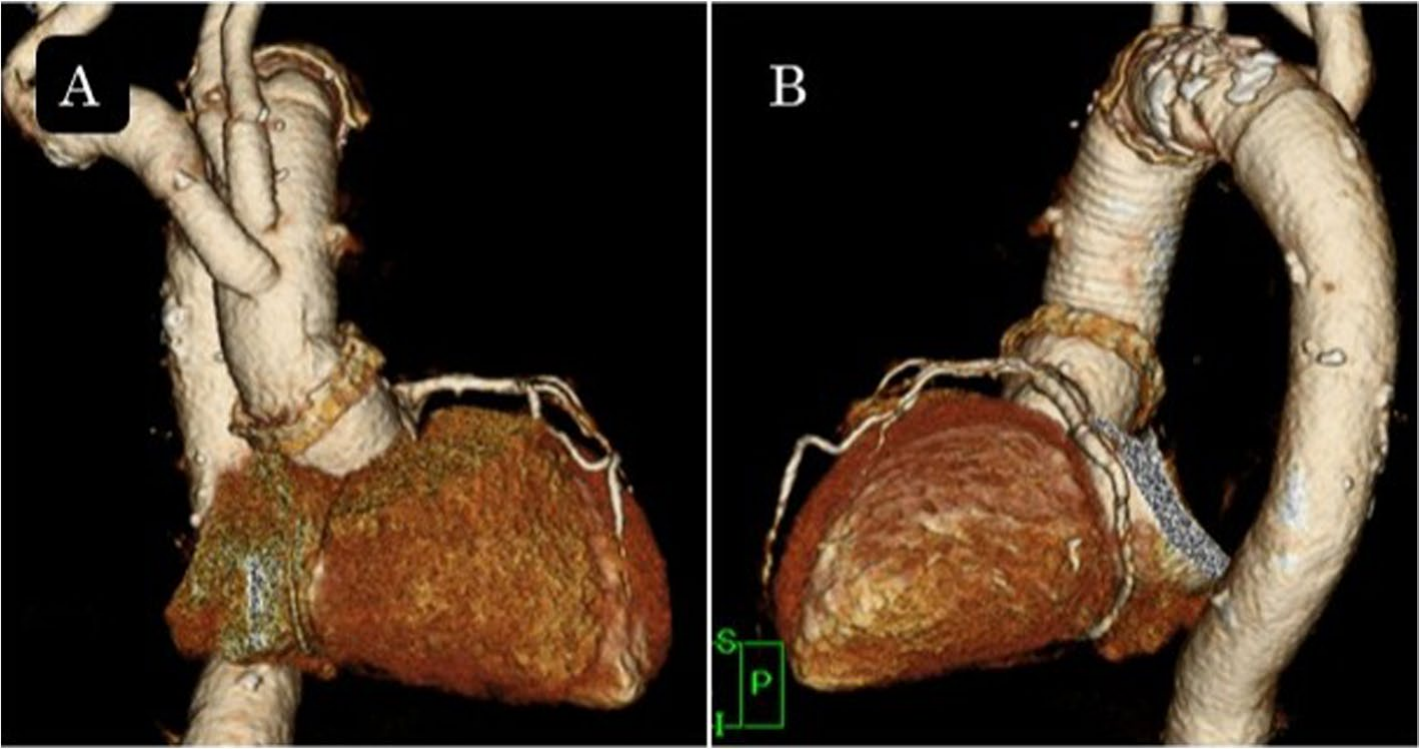
3D computed tomography image taken after the surgery: front view (**A**) and left lateral view (**B**)

A 77-year-old woman who had been on medication for chronic atrial fibrillation, hypertension and hyperlipidemia had sudden back pain and was diagnosed with acute type A aortic dissection on computed tomography and was transferred immediately to our hospital. Because the false lumen from the aortic root to the descending aorta was thrombosed entirely, we opted for conservative therapy rather than emergent surgery. Computed tomography for follow-up was performed several times and revealed incidentally a single coronary artery when electrocardiogram-gated computed tomography was taken for a precise assessment of the aortic root. The images revealed the artery arising from the left sinus of Valsalva and no ostium at the right sinus. The artery continuing from the left circumflex artery at the left atrioventricular groove ran continuously through the right side groove and perfused the right ventricle (Fig. 1). The patient was hospitalized for 5 weeks uneventfully and transferred to the previous hospital for continuing rehabilitation. One month later, she experienced chest pain, and computed tomography at the hospital revealed that the false lumen had thrombosed before becoming patent from the ascending aorta to the proximal aortic arch (Fig. 2). The patient was transferred immediately to our department again and underwent an urgent operation involving graft replacement of the ascending aorta and aortic arch. The computed tomography performed during the previous admission showed no severe stenosis or occlusive lesions in the coronary arteries, and there was no coronary artery connection between the aorta and pulmonary artery that could pose a risk for cardiac ischemia. Therefore, we did not plan for coronary revascularization. Cardiopulmonary bypass was established with a graft anastomosed to the right axillary artery and a venous cannula inserted in the right atrium through a median sternotomy. In the operative findings on the aortic root after transection following aortic cross clamp, there was only one ostium of the coronary artery at the left sinus of Valsalva and no ostium at the right sinus. All antegrade cold-blood cardioplegia solutions were selectively injected into the ostium at the left sinus, and cardiac arrest was achieved, followed by retrograde administration of cardioplegia through the coronary sinus. We employed the administration intermittently after that. The false lumen of the right and non-coronary sinuses of Valsalva were glued with Bioglue, and Teflon felt strips inside and outside were attached for reapproximation of the proximal aortic stump. When systemic cooling to 26 °C at the rectum was achieved, circulatory arrest and selective cerebral perfusion were employed. We performed distal aortic anastomosis before resumption of cardiopulmonary bypass through the side branch of the graft and proximal anastomosis followed by release of the aortic clamp. The cervical branches were reconstructed, and selective cerebral perfusion was ceased sequentially. Weaning from the cardiopulmonary bypass after systemic warming to 36 °C was uneventful. No remarkable complications occurred postoperatively, and she was transferred to the previous hospital for rehabilitation on the 14th day after surgery. Postoperative computed tomography revealed no remarkable problems (Fig. 3).

## Discussion and conclusions

Single coronary artery is a rare anomaly in which the coronary artery ostium is solitary and located at either the left or right sinus of Valsalva, and another coronary artery arises from anywhere in the artery. Lipton et al. reported the classification of this anomaly, which is initially divided into 2 types, “R” or “L”, according to the ostium at either the left or right sinus of Valsalva [5]. The next step is to classify them into 3 types, “I”, “II” or “III”, according to the course of the artery arising from the artery with an ostium at the sinus of Valsalva. The type I artery originates from the distal portion of the right or left circumflex artery at the atrioventricular groove. The type II artery arises from the proximal portion of another artery and runs near the root of the great vessels. In type III, the left anterior descending and circumflex arteries arise from the proximal portion of the right coronary artery with an ostium at the right sinus. Furthermore, type II is divided into 3 types according to the course of the artery arising from another artery. Running in the “A”nterior of the pulmonary trunk is type “A”, “B”etween the great vessels is type “B”, and running in the “P”osterior of the aorta is type “P”. Therefore, the classification is represented, for example, as “L I”, “R II-B”, “L II-P”, or “R III”. In this case, the origin of the coronary artery is at the left sinus of Valsalva, and another artery (right coronary artery without an ostium at the sinus of Valsalva) is continuing from the left circumflex artery. So this is classified as “L I” according to the classification. This disease does not have an operative indication per se but is occasionally reported to cause coronary ischemia, especially type “B”, when the artery between the great vessels is squeezed by the expanding vessels upon exertion.

Type A aortic dissection occurring in patients with a single coronary artery is rare. As far as we explored PubMed, only 3 cases have been reported previously [6–8]. Although it is unclear whether this coronary anomaly is linked to aortic fragility, two of these three cases had connective tissue disorders, specifically Marfan syndrome and Turner’s syndrome respectively. There have been report of patient with a quadricuspid aortic valve who underwent surgical repair for ascending aortic dilation [9]. Thus, there is a possibility that this coronary anomaly is related with aortic pathology.

In all patients with type A aortic dissection mentioned earlier [6–8], surgery was performed emergently, and the anomaly of the coronary artery was recognized for the first time in the operating room. In this case, the operation was performed in the chronic phase of aortic dissection, and the anomaly was recognized preoperatively on electrocardiogram-gated computed tomography taken during conservative therapy. Therefore, we were able to assess the coronary ischemia, allowing us to administer the cardioplegia solution smoothly through only one coronary artery orifice, without wasting time searching for the other orifice. Images of the aortic root on non-electrocardiogram-gated computed tomography are frequently not clear because of motion artifacts of the heart. We recognized the anomaly on follow-up computed tomography gated with an electrocardiogram rather than on non-gated computed tomography immediately after onset. It might be important to notify doctors treating acute patients in an emergency department that electrocardiogram-gated computed tomography should be considered when aorta-related disease is suspected because it may be possible to assess the aortic root precisely, and findings of regions such as this anomaly have a massive effect on the surgical strategy.

We experienced a patient with a dissecting aortic aneurysm with an anomalous single coronary artery and successfully underwent graft replacement of the affected aorta. Electrocardiogra-gated computed tomography was useful for detecting the anomaly. Only 3 patients with a single coronary artery suffering from aortic dissection have been reported previously.

## Data Availability

The datasets used during the current study are available from the corresponding author on reasonable request.
